# Comparing the Effectiveness of Radiofrequency Ablation and Intra-articular Steroid Injections in the Management of Knee Osteoarthritis: A Systematic Review

**DOI:** 10.7759/cureus.104652

**Published:** 2026-03-04

**Authors:** Odiaka M Anombem, Jacob E Oliver, Joshua Kilmer, Robert Tompkins, Christian Ferrer

**Affiliations:** 1 Rural Family Medicine, University of Texas Health Science Center, Tyler, USA

**Keywords:** injection, knee, osteoarthritis, radiofrequency, steroid

## Abstract

Knee osteoarthritis is a progressive joint disorder that causes chronic pain, disability, significant impairment in quality of life, and a substantial burden on the health care system. Although conservative approaches, like physical therapy and pharmacological interventions, provide the initial treatment for knee osteoarthritis, patients with inadequate pain relief and other refractory symptoms often require other minimally invasive therapies, such as intra-articular steroid injection (IASI) or genicular nerve radiofrequency ablation (GNRFA).

The purpose of this systematic review is to comprehensively compare the effectiveness of GNRFA and IASI in the treatment of knee osteoarthritis. Using the PRISMA 2020 guidelines, this systematic review searched multiple databases, including PubMed, Web of Science, Scilit, and the Cochrane Library, for articles published between March 2015 and 2025. After screening, four randomized controlled trials, involving 379 patients, met the inclusion criteria and were included in our systematic review.

Results demonstrated that both interventions showed significant improvements in pain and also improved function compared to baseline. IASI provided superior pain relief and functional improvement compared to GNRFA at one week post-intervention. At three months, IASI also showed a statistically superior improvement in knee stiffness compared with GNRFA. However, GNRFA consistently showed superior, more sustained benefits from one to six months post-intervention, including greater pain reduction, improved function, decreased non-opioid analgesic use, and higher patient satisfaction. Effect sizes for pain favoring GNRFA progressed from small at one month (SMD: -0.398) to large at six months (SMD: -1.504), reflecting increasing GNRFA superiority over time. At the end of six months, 22% of patients in the GNRFA group reported complete pain relief, compared with only 4% in the IASI group. Both modalities demonstrated equivalent safety outcomes, with minimal adverse effects.

These findings suggest that, while IASI offers rapid symptomatic relief suitable for patients requiring immediate pain control or those with prominent joint stiffness, GNRFA provides superior, sustained therapeutic benefits for patients seeking durable pain control and functional improvement. Treatment selection for patients should be individualized and achieved through shared decision-making, considering patient preferences, symptom patterns, and desired duration of therapeutic benefit. Future research should explore longer-term comparative effectiveness studies beyond six months, identify patient subgroups most likely to benefit from each intervention, and evaluate combination or sequential treatment strategies to optimize knee osteoarthritis management.

## Introduction and background

Knee osteoarthritis is a degenerative joint disease that causes chronic pain and disability worldwide, affecting millions of individuals globally [1]. The prevalence of knee osteoarthritis increases significantly with age, with studies showing that, in the United States, about 10% of men and 13% of women aged 60 years or older experience symptomatic knee osteoarthritis [2]. Osteoarthritis is characterized by progressive cartilage degeneration, subchondral bone changes, inflammation, and the formation of osteophytes, leading to clinical manifestations such as joint pain, stiffness, and functional impairment [3]. Knee osteoarthritis is a major contributor to disability in older adults and places a considerable burden on both the health care system and economy. This burden includes not only the direct medical costs, but also indirect costs associated with work absenteeism and reduced productivity [4].

The initial management of knee osteoarthritis usually involves conservative approaches, such as weight management, physical therapy, and pharmacological pain control with non-steroidal anti-inflammatory drugs (NSAIDs) and other analgesic agents [5]. However, for patients who experience inadequate pain relief from these conservative treatments but wish to delay or avoid total knee replacement surgery, there are other minimally invasive treatment options, such as intra-articular steroid injections (IASIs) and genicular nerve radiofrequency ablation (GNRFA) [6]. These minimally invasive therapies are particularly relevant for providing patients with symptomatic pain relief and bridging the gap between conservative management and definitive surgical intervention [6].

IASIs are widely used and remain one of the most commonly performed procedures in the management of knee osteoarthritis [7]. The mechanism of action involves the anti-inflammatory effects of corticosteroids, which include reducing the inflammatory cells within the joint, decreasing lysosomal enzyme release, and inhibiting pro-inflammatory cytokines. These reduce synovial inflammation, reduce pain, and improve joint functionality [8]. However, repeated corticosteroid injections have been associated with chondrotoxicity and progressive cartilage degeneration [8]. Generally, IASI is considered a low-cost, office-based procedure that usually does not require anesthesia [9]. It can be performed with or without ultrasound or fluoroscopic guidance. However, using ultrasound or fluoroscopy will often lead to a higher overall cost [9].

GNRFA is another minimally invasive option for treating knee osteoarthritis. The knee joint capsule is innervated by genicular nerves, which play a central role in transmitting the pain sensation of the knee [10]. The genicular nerves originate from branches of three major nerves, namely the femoral nerve, the sciatic nerve, and the obturator nerve [11]. The tibial nerve gives rise to the superomedial and inferomedial genicular nerves, while the common peroneal nerve gives rise to the superolateral genicular nerve [11]. The conventional GNRFA technique targets the superolateral, superomedial, and inferomedial genicular nerves [12]. GNRFA aims to reduce pain by selectively targeting genicular nerves and interrupting pain-signal transmission. This prevents patients from experiencing knee pain associated with osteoarthritis. This treatment is typically performed under imaging guidance, by placing electrodes at specific anatomical landmarks that are adjacent to the targeted genicular nerve [10]. Controlled thermal energy is then generated in the targeted nerves, which disrupts the pain signaling pathway. This results in the reduction of perceived knee pain in patients with osteoarthritis [6].

GNRFA is a more costly procedure than IASI, as it is typically performed in an ambulatory surgical center or operating room under fluoroscopic guidance [13]. It is also usually performed using a local anesthetic, and sometimes with mild sedation and monitored anesthesia care. The procedure usually requires sterile facility resources and specialized radiofrequency equipment [14]. The combination of the specialized setting, anesthesia support, and advanced equipment makes GNRFA more expensive than IASI.

There are several radiofrequency techniques used in the management of knee osteoarthritis, such as conventional radiofrequency ablation, cooled radiofrequency ablation, and pulsed radiofrequency [11]. In conventional radiofrequency ablation, a continuous high-frequency electrical current is delivered to the electrodes. This results in the generation of localized thermal energy, which produces coagulative necrosis of the targeted sensory nerve and interrupts pain signal transmission [15]. Cooled radiofrequency ablation similarly uses a continuous high-frequency electrical current delivered to the electrodes, but the electrode tip is cooled with a circulating fluid. This enables higher power delivery and allows a larger tissue volume to be targeted with this procedure [15]. In contrast, pulsed radiofrequency applies the current intermittently rather than continuously, and this causes neuromodulation of the adjacent nerve, and does not cause coagulative necrosis [16]. A comparative study demonstrated similar pain outcomes among conventional radiofrequency ablation, cooled radiofrequency ablation, and pulsed radiofrequency in the treatment of patients with knee osteoarthritis [17].

Despite the growing use of GNRFA for knee osteoarthritis, limited evidence exists on how its efficacy compares with more established treatments, like IASI. This systematic review aims to provide a robust comparison of GNRFA and IASI across several key outcome domains, which include pain reduction, functional improvement, patient satisfaction, analgesic medication use, and safety profile. While a previous review compared these two treatment modalities [18], this review aims to perform a more comprehensive analysis by conducting a broader database search, screening a larger volume of studies, and obtaining more outcome data. The findings in this review are intended to strengthen and broaden the existing body of knowledge and to provide evidence-based guidance to assist clinicians and patients in making informed treatment decisions for knee osteoarthritis management.

## Review

Methodology

Review Protocol

To ensure a systematic review process, the study was conducted in accordance with the Preferred Reporting Items for Systematic Reviews and Meta-Analyses (PRISMA) 2020 guidelines [19]. To maintain transparency and consistency, the study protocol was developed prior to the study’s initiation. The study protocol was also registered in PROSPERO, with ID CRD420251141128.

Search Strategy

A comprehensive literature search was conducted across four electronic databases: PubMed, Web of Science, Scilit, and the Cochrane Library. These databases were searched for relevant studies published between March 1, 2015, and March 1, 2025. The search strategy employed the following combination of keywords: “knee,” “osteoarthritis,” “radiofrequency,” “steroid,” and “injection.”

To optimize retrieval across different database platforms, comprehensive search strategies were constructed using Boolean operators (AND, OR) and PubMed MeSH terms. Each search query was adapted to the specific syntax and indexing structure of the respective database search engine to ensure maximum coverage of relevant literature. The search strategies used for each database are presented in Table 1.

**Table 1:** Search Strategy for All Databases

S. No.	Database	Search Query	Number of Articles Found	Filters Applied
1.	PubMed	(("knee"[MeSH Terms] OR "knee"[All Fields] OR "knee joint"[MeSH Terms] OR ("knee"[All Fields] AND "joint"[All Fields]) OR "knee joint"[All Fields]) AND ("osteoarthritis"[MeSH Terms] OR "osteoarthritis"[All Fields] OR "osteoarthritides"[All Fields]) AND ("radiofrequencies"[All Fields] OR "radiofrequency"[All Fields] OR "radiofrequent"[All Fields]) AND ("steroidal"[All Fields] OR "steroidals"[All Fields] OR "steroidic"[All Fields] OR "steroids"[Supplementary Concept] OR "steroids"[All Fields] OR "steroid"[All Fields] OR "steroids"[MeSH Terms]) AND ("inject"[All Fields] OR "injectability"[All Fields] OR "injectant"[All Fields] OR "injectants"[All Fields] OR "injectate"[All Fields] OR "injectates"[All Fields] OR "injected"[All Fields] OR "injectible"[All Fields] OR "injectibles"[All Fields] OR "injecting"[All Fields] OR "injections"[MeSH Terms] OR "injections"[All Fields] OR "injectable"[All Fields] OR "injectables"[All Fields] OR "injection"[All Fields] OR "injects"[All Fields]) AND 2015/03/01:2025/03/01[Date - Publication]) AND (english[Filter])	22	None
2.	Web of Science	TS=("Knee" OR "Knee joint") AND TS=("Osteoarthritis" OR "Osteoarthritides") AND TS=("Radiofrequency" OR "Radiofrequencies" OR "Radiofrequent") AND TS=("Steroid" OR "Steroidic" OR "Steroids" OR "Steroidal") AND TS=("Injection" OR "Injectants" OR "Inject" OR "injected" OR "Injectables" OR "Injects") AND LA=English	17	March 1, 2015 - March 1, 2025
3.	Scilit	(Knee OR Knees) AND (Osteoarthritis) AND (Radiofrequency OR Radiofrequencies) AND (Steroid OR Steroids) AND (Injection OR Inject OR Injections)	20	English language, March 1, 2015 - March 1, 2025
4.	Cochrane Library	(Knee OR Knee joint) AND (Osteoarthritis" OR Osteoarthritides) AND (Radiofrequencies OR Radiofrequency OR Radiofrequent) AND (Steroid OR Steroidic OR Steroids OR Steroidal) AND (Injection OR Injectants OR Inject OR Injected OR Injectables)	23	English language, March 1, 2015 - March 1, 2025

Eligibility Criteria

Inclusion and exclusion criteria for this study are outlined in Table 2. The inclusion criteria for this review comprised peer-reviewed articles, including randomized controlled trials, non-randomized controlled trials, and observational studies, such as cohort and case-control studies. All research papers were required to be published between March 1, 2015, and March 1, 2025, with osteoarthritis of the knee as the primary pathology under investigation. Eligible studies included adult participants aged 18 years and older, who were diagnosed with knee osteoarthritis based on clinical and/or radiological criteria. Studies were required to examine either GNRFA or IASI as management strategies for knee osteoarthritis, with particular interest in the direct comparison of these two interventions.

**Table 2:** Inclusion and Exclusion Criteria

S. No.	PICO	Inclusion Criteria	Exclusion Criteria
1	Population	Adults (≥18 years) diagnosed with knee osteoarthritis based on clinical and/or radiological criteria. Both sexes.	Patients with other types of arthritis (e.g., rheumatoid arthritis). Non-human studies. Pediatric populations (<18 years).
2	Intervention	Genicular nerve radiofrequency ablation used in the management of knee osteoarthritis.	Radiofrequency ablation applied to joints other than the knee.
3	Comparator	Intra-articular steroid injection used in the management of knee osteoarthritis.	Other intra-articular treatments, such as hyaluronic acid, protein-rich plasma. Also, Intra-articular steroid injection used for joints other than the knee.
4	Outcomes	The primary outcome of this review is pain reduction following intervention, which will be evaluated using standardized pain assessment tools such as the Visual Analog Scale and the Numeric Rating Scale. Secondary outcomes include functional improvement and the duration of therapeutic effect, assessed using validated measures such as the Western Ontario and McMaster Universities Osteoarthritis Index, the Global Perceived Effect Scale, and the Oxford Knee Scale.	Studies reporting outcomes unrelated to pain reduction or functional improvement.
5	Study design	This review will consider research studies that evaluate and compare relevant interventions, including randomized controlled trials, non-randomized controlled trials, and observational studies such as cohort and case-control studies.	This review will exclude case reports, case series, opinion pieces, letters, and conference summaries with unavailable full articles. Also, non-original articles such as systematic reviews and meta-analyses will not be included in the analysis.
6	Year of publication	Research papers published between March 1, 2015, and March 1, 2025.	Research papers published before March 1, 2015, or after March 1, 2025.
7	Language	Published in English.	Published in other languages apart from English.

Exclusion criteria encompass non-original research, such as systematic reviews, meta-analyses, opinion pieces, letters, case reports, case series, and conference abstracts without accessible full texts. Studies were also excluded if they did not focus on knee osteoarthritis, involved patients with other forms of arthritis (e.g., rheumatoid arthritis), included non-human subjects, enrolled participants younger than 18 years, applied radiofrequency ablation to joints other than the knee, or evaluated treatment modalities outside of GNRFA and IASI.

Study Selection

All identified articles from all the databases were uploaded to Covidence [20], which is a systematic review management tool. In Covidence, duplicate studies were removed. Then, two authors independently screened the papers by reviewing the titles of the research articles. This was followed by a full-text assessment to determine eligibility. The studies that met the inclusion criteria were selected for the final review. 

Data Extraction

Data extraction was carried out independently by two authors, using a standardized template to ensure consistency. The extracted data comprised various categories, including general study characteristics, such as authors’ names, publication year, and study location. The methodological information included the study design, recruitment methods, and blinding procedures. The participants’ information included baseline demographics and clinical features. The intervention information included the specifics of the interventions administered, such as the type and protocol of the GNRFA or IASI. The reported outcomes included pain scores, functional assessments, quality-of-life metrics, and safety data. Any discrepancies between reviewers were discussed and resolved collaboratively, with a third reviewer consulted when necessary. 

Assessment of Quality and Risk of Bias

The quality and risk of bias of the included studies were systematically evaluated, independently by two authors, using validated assessment tools appropriate to each study design. For randomized controlled trials, the Cochrane Risk of Bias 2 tool was used to assess potential biases across multiple domains [21]. The visual representation of the risk-of-bias assessment findings was created using ROBVIS [22], which is a visualization tool used to summarize risk-of-bias evaluations of the included studies. 

Data Synthesis

The extracted data were imported and analyzed in R (version 4.5.2; R Foundation for Statistical Computing, Vienna, Austria) within RStudio (Version: 2026.01.1+403; RStudio, Inc., Boston, MA, USA), a statistical analysis tool. This was used to calculate the effect sizes and assess for heterogeneity. Effect sizes were calculated as standardized mean differences using Cohen’s d. Heterogeneity was assessed using the I² statistic. Two of the four included studies reported sufficient data in a compatible format to permit quantitative synthesis [23], while the remaining two studies were included in the narrative synthesis. Heterogeneity was present in five of the six planned meta-analytic comparisons. For knee pain assessment, I² ranged from 47.10% at one month to 90.20% at six months. For knee function, heterogeneity ranged from 68.41% at one month to 97.54% at six months. The summary of heterogeneity findings is presented in Tables 3-4. Due to substantial heterogeneity, a meta-analysis was not performed. Instead, a narrative synthesis was conducted, with effect sizes and key findings presented in text and tables to highlight patterns, similarities, and differences across the four included studies.

**Table 3:** Heterogeneity Statistics for the Studies Assessing Knee Pain Outcomes I² = Proportion of Total Variability Attributable to Heterogeneity; Cochran's Q = Test Statistic for heterogeneity; p-value (Q) = p-value for Cochran's Q test.

Studies	Time Point	I^2^	Cochran’s Q	p-value (Q)
Davis et al. [23], Badawy et al. [24]	1 month	47.10%	1.890	0.169
Davis et al. [23], Badawy et al. [24]	3 months	74.77%	3.964	0.0465
Davis et al. [23], Badawy et al. [24]	6 months	90.20%	10.206	0.0014

**Table 4:** Heterogeneity Statistics for the Studies Assessing Knee Function Outcomes I² = Proportion of Total Variability Attributable to Heterogeneity; Cochran's Q = Test Statistic for heterogeneity; p-value (Q) = p-value for Cochran's Q test.

Studies	Time Point	I^2^	Cochran’s Q	p-value (Q)
Davis et al. [23], Badawy et al. [24]	1 month	68.41%	3.165	0.0752
Davis et al. [23], Badawy et al. [24]	3 months	98.07%	51.739	<0.0001
Davis et al. [23], Badawy et al. [24]	6 months	97.54%	40.609	<0.0001

Results

Study Selection Process

**Figure 1:**
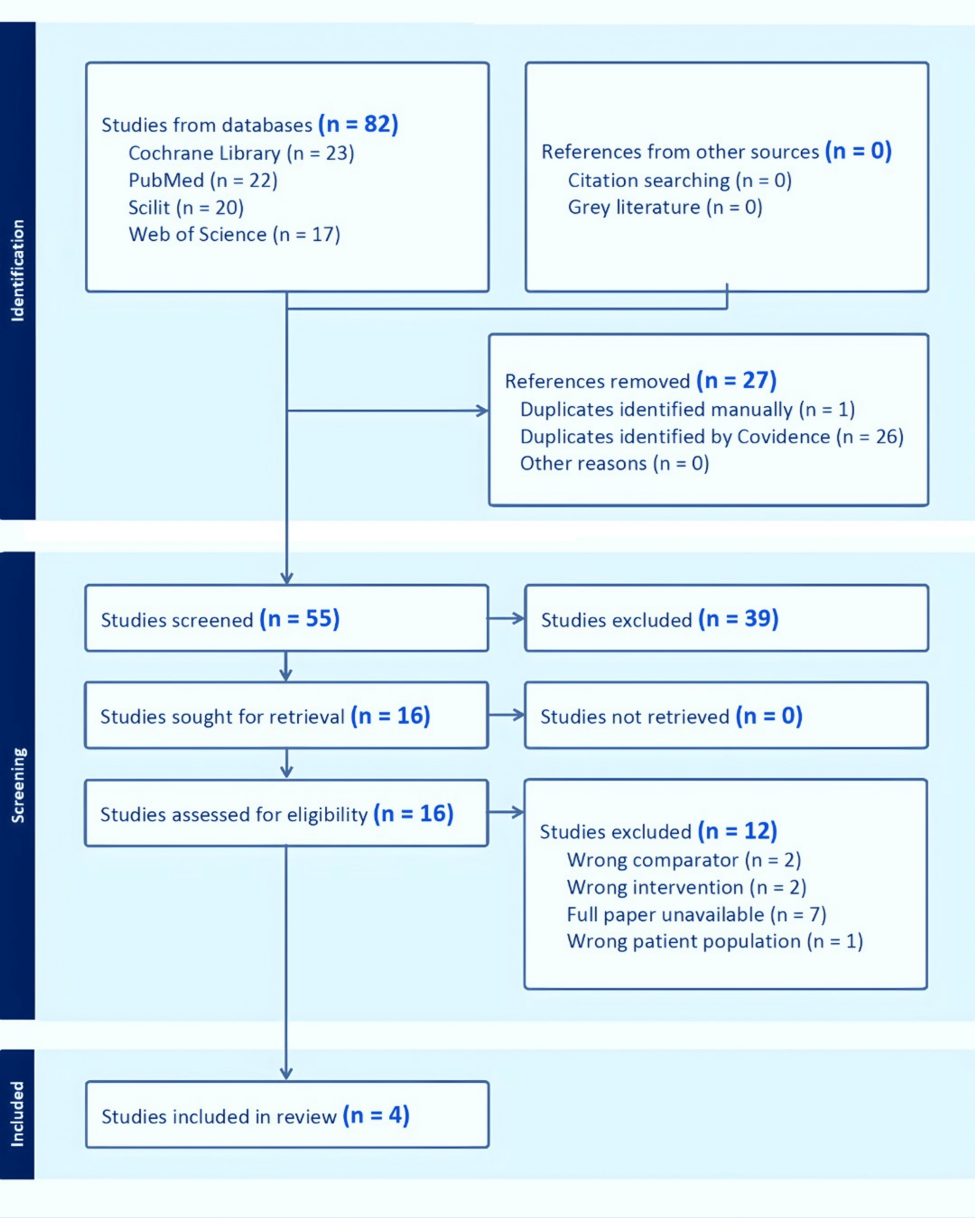
PRISMA Flow Diagram Illustrating the Study Selection Process PRISMA: Preferred Reporting Items for Systematic Reviews and Meta-Analyses

A total of 82 studies were identified from PubMed (n = 22), Web of Science (n = 17), Scilit (n = 20), and the Cochrane Library (n = 23). Studies were uploaded, screened, and data were extracted using Covidence [20]. Of these, 27 studies were identified as duplicates and removed, leaving 55 studies for screening. These studies were further screened based on their titles and abstracts, and 39 were excluded for failing to meet the inclusion criteria. The remaining 16 studies underwent full-text review, of which four randomized controlled trials met all eligibility criteria and were included in the systematic review. Figure 1, the PRISMA flow diagram, clearly describes the study selection process.

Study Characteristics and Demographics

All four included studies were randomized controlled trials comparing GNRFA with IASI in patients with knee osteoarthritis [23-26]. In one study [26], the methodology section described the study as a randomized controlled trial, whereas the study design section referred to it as a retrospective comparative study. However, the reported methods indicate that participants were randomized, blinding was implemented, and outcomes were assessed over a structured six-month follow-up period, features that are consistent with a randomized controlled trial. The sample sizes of included studies ranged from a minimum of 58 to a maximum of 151. The total number of subjects was 379, with 208 patients receiving radiofrequency treatment and 171 patients receiving IASI. The mean age of participants ranged from 45.0 to 64.5 years, and the percentage of female participants ranged from 57.8% to 78.1%. Studies were conducted across various countries, including the United States [23], Turkey [25], China [26], and Egypt [24]. The selected studies used cooled radiofrequency ablation [23], conventional radiofrequency ablation [24-26], and pulsed radiofrequency [26]. In all four studies, radiofrequency ablation and IASI were compared using standardized scoring systems, such as the Numeric Rating Scale (NRS), Oxford Knee Score (OKS), Global Perceived Effect (GPE), Visual Analog Scale (VAS), and Western Ontario and McMaster Universities Osteoarthritis Index (WOMAC). Table 5 summarizes the characteristics of the included studies.

**Table 5:** Characteristics of Selected Studies RFA: Radiofrequency Ablation; IASI: Intra-articular Steroid Injection; RF: Radiofrequency; RCT: Randomized Controlled Trial; NRS: Numeric Rating Scale; OKS: Oxford Knee Score; GPE: Global Perceived Effect; VAS: Visual Analog Scale; WOMAC: Western Ontario and McMaster Universities Osteoarthritis Index

No.	Study (Author and Year)	Country	Study Design	Subject Population	Mean Age (Years)	Duration of Study (Months)	Interventions	Comparators	Outcomes Measured
1	Davis et al. (2018) [23]	United States	RCT	151	64.5	6	Cooled RFA,	IASI: Betamethasone, Methylprednisolone, Triamcinolone	NRS, OKS, GPE, Pain Medication Use, Adverse Effects
2	Sari et al. (2018) [25]	Turkey	RCT	73	64	3	Conventional RFA	IASI: Betamethasone	VAS, WOMAC, Adverse Effects
3	Hong et al. (2020) [26]	China	RCT	97	60.5	6	Conventional RFA, Pulsed intra-articular RF	IASI: Betamethasone	NRS, OKS, GPE, Adverse Effects
4	Badawy et al. (2021) [24]	Egypt	RCT	58	45	6	Conventional RFA	IASI: Methylprednisolone	WOMAC, VAS, Patient Satisfaction, Adverse Effects

Pain Outcomes

Across all four included studies [23-26], both GNRFA and IASI demonstrated significant reductions in knee pain severity in patients with osteoarthritis, compared with baseline. To assess pain severity during follow-up visits in the selected studies, various scales were used, including the NRS, VAS, and the WOMAC pain subscale. The summary of effect sizes for knee pain is presented in Table 6.

**Table 6:** Standardized Mean Differences for Knee Pain at One, Three, and Six Months SMD, Standardized Mean Difference (Effect Size); CI, Confidence Interval

Studies	Time Point	SMD (Effect Size)	95% CI	p-value
Davis et al. [23]	1 month	-0.398	-0.737 to -0.058	0.0216
Badawy et al. [24]	1 month	-0.844	-1.381 to -0.306	0.0021
Davis et al. [23]	3 months	-1.136	-1.503 to -0.770	<0.0001
Badawy et al. [24]	3 months	-0.488	-1.011 to 0.034	0.0670
Davis et al. [23]	6 months	-1.504	-1.901 to -1.108	<0.0001
Badawy et al. [24]	6 months	-0.437	-0.958 to 0.084	0.0958

At one week post-treatment, Hong et al. [26] found that pain relief was significantly greater in the IASI group than the GNRFA group, as measured by the NRS (p < 0.05).

At one month, SMD values were -0.398 (95% CI: -0.737 to -0.058; p = 0.022) for Davis et al. [23] and -0.844 (95% CI: -1.381 to -0.306; p = 0.002) for Badawy et al. [24]. Davis et al. [23] demonstrated a small effect favoring GNRFA, while Badawy et al. [24] demonstrated a large effect favoring GNRFA, both of which were statistically significant. Also, at one-month post-treatment, three studies [23-25] reported that participants treated with GNRFA experienced a significantly greater pain reduction compared with those receiving IASI. In the study by Davis et al. [23], the mean NRS pain score in the GNRFA group decreased from 7.3 at baseline to 3.0 at one month, whereas the IASI group showed a reduction from 7.2 to 3.9 (p = 0.025). Sarı et al. [25] demonstrated that, at one month, GNRFA produced a significantly greater reduction in VAS pain scores compared with IASI (p < 0.001). Similarly, in the study by Badawy et al. [24], the WOMAC pain score in the GNRFA group decreased from 11.7 at baseline to 5.9 at one month, whereas the IASI group showed a decrease from 11.34 at baseline to 7.0 at one month (p = 0.003).

At two months post-treatment, Badawy et al. [24] showed that GNRFA yielded significantly better pain relief outcomes than IASI. There was a decrease in WOMAC pain score in the GNRFA group, from 11.72 at baseline to 5.8 at two months, whereas the IASI group showed a decrease in WOMAC pain score, from 11.34 at baseline to 7.17 at two months (p = 0.003). While GNRFA continued to show sustained pain relief over time, the pain reduction provided by IASI had begun to diminish.

At three months, the SMD values were -1.136 (95% CI: -1.503 to -0.770; p < 0.0001) for Davis et al. [23] and -0.488 (95% CI: -1.011 to 0.034; p = 0.067) for Badawy et al. [24]. Davis et al. [23] demonstrated a large effect favoring GNRFA, which was statistically significant (p < 0.0001), while Badawy et al. [24] showed a small effect, also favoring GNRFA, though the result was not statistically significant (p = 0.067). Also, at three months post-treatment, two studies [23,25] showed that the GNRFA group continued to provide significantly greater pain relief compared with IASI. Davis et al. [23] showed that the NRS pain score declined from 7.3 at baseline to 2.8 at three months in the GNRFA group, versus 7.2 at baseline to 5.2 at three months in the IASI group (p < 0.0001). GNRFA continued to demonstrate prolonged pain relief, in contrast to IASI, where the degree of pain relief continued to wane over time. At the three-month follow-up, Sarı et al. [25] reported that GNRFA achieved a markedly greater decrease in VAS pain scores compared with IASI (p < 0.001).

At six months, the SMD values were -1.504 (95% CI: -1.901 to -1.108; p < 0.0001) for Davis et al. [23] and -0.437 (95% CI: -0.958 to 0.084; p = 0.096) for Badawy et al. [24]. Davis et al. [23] demonstrated a large effect favoring GNRFA, which was statistically significant (p < 0.0001), while Badawy et al. [24] showed a small effect, also favoring GNRFA, though the result was not statistically significant (p = 0.096). Also, at six months, Davis et al. [23] reported superior pain outcomes in the GNRFA group compared with IASI. In this study [23], the mean NRS pain score declined from 7.3 at baseline to 2.5 at six months in the GNRFA group, whereas the IASI group decreased from 7.2 at baseline to 5.9 at six months (p < 0.0001). GNRFA continued to provide sustained pain relief, whereas IASI has shown a steady reduction in pain reduction as time has progressed.

Davis et al. [23] found that 74.1% of the participants in the GNRFA group achieved at least a 50% reduction in knee pain at six months, compared with 16.2% in the IASI group (p < 0.0001). Furthermore, 22% of the GNRFA patients reported having no pain at six months, compared with only 4% of the IASI patients (p = 0.0026). Interestingly, none of the GNRFA subjects experienced worsening pain, whereas 15% of the IASI participants experienced worsening pain (p = 0.0024).

Functional Outcomes

Findings from all four selected studies [23-26] indicated that GNRFA and IASI each produced a statistically significant enhancement in knee function, compared with baseline measures. Knee function was assessed in the selected studies using two scales, including the OKS and the WOMAC function subscale. The summary of effect sizes for knee function is presented in Table 7.

**Table 7:** Standardized Mean Differences for Knee Function at One, Three, and Six Months SMD, Standardized Mean Difference (Effect Size); CI, Confidence Interval

Studies	Time Point	SMD (Effect Size)	95% CI	p-value
Davis et al. [23]	1 month	0.4381	0.098 to 0.778	0.0116
Badawy et al. [24]	1 month	-0.122	-0.638 to 0.393	0.6418
Davis et al. [23]	3 months	1.251	0.879 to 1.622	<0.0001
Badawy et al. [24]	3 months	-1.217	-1.778 to -0.657	<0.0001
Davis et al. [23]	6 months	1.53	1.130 to 1.929	<0.0001
Badawy et al. [24]	6 months	-0.62	-1.147 to -0.093	0.021

One week after treatment, Hong et al. [26] reported that IASI produced a statistically superior improvement in knee function compared with the GNRFA group, as assessed by the OKS (p < 0.001). 

At one month, the SMD values were 0.438 (95% CI: 0.098 to 0.778; p = 0.012) for Davis et al. [23] and -0.122 (95% CI: -0.638 to 0.393; p = 0.642) for Badawy et al. [24]. Davis et al. [23] demonstrated a small effect favoring GNRFA, which was statistically significant (p = 0.012), while Badawy et al. [24] showed a negligible effect, which was not statistically significant (p = 0.642). Also, at one month after treatment, two studies [23,25] reported that the GNRFA group demonstrated significantly greater improvement in knee function than the IASI group. Davis et al. [23] showed that the GNRFA group improved in knee function, with the mean OKS increasing from 16.7 at baseline to 33.3 at one month, whereas the IASI group improved from 16.9 at baseline to 29.4 at one month (p = 0.004). Similarly, Sarı et al. [25] observed that the GNRFA group’s WOMAC function score decreased from 41 at baseline to 24 at one month, whereas the IASI group’s score decreased from 36 at baseline to 29.5 at one month (p = 0.003). 

Two months after treatment, Badawy et al. [24] showed that the GNRFA group’s WOMAC function score decreased from 40.76 at baseline to 23.28, while the IASI group’s score declined from 40.21 to 25.31 (p = 0.002), demonstrating that the GNRFA group achieved greater improvement in knee function than the IASI group.

At three months, the SMD values were 1.251 (95% CI: 0.879 to 1.622; p < 0.0001) for Davis et al. [23] and -1.217 (95% CI: -1.778 to -0.657; p < 0.0001) for Badawy et al. [24]. Davis et al. [23] demonstrated a large effect favoring GNRFA, which was statistically significant (p < 0.0001), while Badawy et al. [24] also demonstrated a large effect favoring GNRFA, which was statistically significant (p < 0.0001). The opposing signs reflect differences in scoring direction between the OKS and WOMAC function subscale, not a disagreement in outcome direction. At three months after treatment, two studies [23,24] showed that patients in the GNRFA group experienced significantly greater improvement in knee function than those in the IASI group. Davis et al. [23] reported that at three months, the GNRFA group showed greater improvement in OKS, increasing from 16.7 at baseline to 34.6 at three months, compared with an increase from 16.9 at baseline to 24.6 at three months in the IASI group (p < 0.0001). Similarly, Badawy et al. [24] demonstrated that the GNRFA group’s WOMAC function score decreased from 40.76 at baseline to 28.28, whereas the IASI group’s score declined from 40.21 to 32.48 (p = 0.001).

At six months, the SMD values were 1.530 (95% CI: 1.130 to 1.929; p < 0.0001) for Davis et al. [23] and -0.620 (95% CI: -1.147 to -0.093; p = 0.021) for Badawy et al. [24]. Davis et al. [23] demonstrated a large effect favoring GNRFA, which was statistically significant (p < 0.0001), while Badawy et al. [24] also demonstrated a moderate effect favoring GNRFA, which was statistically significant (p = 0.021). The opposing signs reflect differences in scoring direction between the OKS and WOMAC function subscale, not a disagreement in outcome direction. At six months post-treatment, both Davis et al. [23] and Badawy et al. [24] reported that GNRFA resulted in markedly better functional outcomes than IASI. In the study by Davis et al. [23], the GNRFA group showed a further improvement in OKS, increasing from 16.7 at baseline to 35.7 at six months, while the IASI group increased from 16.9 at baseline to 22.4 at six months (p < 0.0001). Similarly, Badawy et al. [24] found that at six months, the GNRFA group’s WOMAC function score decreased from 40.76 at baseline to 32.59, compared with a decline from 40.21 to 35.31 in the IASI group (p = 0.008).

Patient Satisfaction

At one month, two papers [23,26] showed that there was no statistical difference in patient satisfaction between the GNRFA and IASI treatment groups (p = 0.1).

At three months, two papers [23,26] showed greater patient satisfaction in the GNRFA group than in the IASI group. Davis et al. [23] revealed that 80% of participants in the GNRFA group reported improvement in their osteoarthritis, while only 31% of the IASI group reported improvement. Hong et al. [26] showed that GNRFA was statistically superior in the GPE compared with the IASI group (p < 0.05).

Six-month findings from two studies [23,26] consistently favored GNRFA, showing higher levels of patient satisfaction than IASI. In the study by Davis et al. [23], 91% of GNRFA patients reported improvement, whereas just 24% of the IASI group reported improvement. Hong et al. [26] also found GNRFA superior, with significantly better GPE outcomes (p < 0.05).

Analgesic Use

In the study by Davis et al. [23], participants reported their opioid or morphine-equivalent use during the study. Participants in both the GNRFA and IASI groups showed no significant difference in opioid use for pain relief compared with their baseline use.

For non-opioid use, at three months, the GNRFA group showed a greater, statistically significant reduction in non-opioid use compared with IASI (p = 0.03). The difference between the two groups became more pronounced at six months, with the GNRFA group using less non-opioid medication, while the IASI group showed an increase in non-opioid analgesic use (p = 0.02) [23]. 

Safety Outcomes

In the study by Davis et al. [23], the GNRFA group reported fewer adverse events (AEs) and severe adverse events (SAEs) than the IASI group. A total of 61 AEs were reported in 34 GNRFA subjects, and 65 AEs were reported in 30 IASI subjects. Most of the AEs were concluded to be unrelated to the study intervention. Examples of unrelated AEs in the GNRFA group included asthma exacerbation and pyelonephritis [23], while some unrelated AEs in the IASI group included nausea, vomiting, and abdominal pain [23]. The total number of SAEs was four in three GNRFA subjects and eight in seven IASI subjects. None of the SAEs were judged to be related to the study treatment [23].

In one study [26], minor periprocedural pain occurred in one patient in the IASI group and two patients in the GNRFA group, with no SAEs observed. In two studies [24,25], no AEs or complications were reported in either the GNRFA or IASI group. Table 8 summarizes the overall results.

**Table 8:** Comparison of Outcomes Between Genicular Nerve Radiofrequency Ablation (GNRFA) and Intra-articular Steroid Injections (IASI) in Knee Osteoarthritis NRS: Numeric Rating Scale; VAS: Visual Analog Scale; WOMAC: Western Ontario and McMaster Universities Osteoarthritis Index; OKS: Oxford Knee Score; GPE: Global Perceived Effect; GNRFA: Genicular Nerve Radiofrequency Ablation; IASI: Intra-articular Steroid Injection

Outcome Measure	Measure Used	Time Point	GNRFA	IASI	Superior Intervention	p-value	Studies
Pain	NRS	1 Week	Moderate Reduction	Greater Reduction	IASI	0.020	Hong et al. [26]
Pain	NRS, VAS, WOMAC Pain	1 Month	Greater Reduction	Moderate Reduction	GNRFA	<0.001 - 0.025	Davis et al. [23], Sari et al. [25], Badawy et al. [24]
Pain	NRS, VAS, WOMAC Pain	3 Months	Sustained Reduction	Declining Effect	GNRFA	<0.001 - 0.001	Davis et al. [23], Sari et al. [25], Badawy et al. [24]
Pain	NRS, VAS	6 Months	Sustained Reduction	Near Baseline	GNRFA	<0.001 - 0.05	Davis et al. [23], Badawy et al. [24]
Functional Improvement	OKS	1 Week	Moderate	Greater	IASI	<0.01	Hong et al. [26]
Functional Improvement	OKS, WOMAC Function	1 Month	Greater	Moderate	GNRFA	0.003 - 0.004	Davis et al. [23], Sari et al. [25]
Functional Improvement	OKS, WOMAC Function	3 Months	Sustained	Declining	GNRFA	<0.0001 - 0.001	Davis et al. [23], Badawy et al. [24]
Functional Improvement	OKS, WOMAC Function	6 Months	Sustained	Declining	GNRFA	<0.0001 - 0.008	Davis et al. [23], Badawy et al. [24]
Patient Satisfaction	GPE	1 Month	Similar	Similar	Neither	0.1	Davis et al. [23], Hong et al. [26]
Patient Satisfaction	GPE, Self-report	3 Months	Higher Satisfaction	Moderate Satisfaction	GNRFA	<0.05	Davis et al. [23], Hong et al. [26]
Patient Satisfaction	GPE, Self-report	6 Months	Higher Satisfaction	Moderate Satisfaction	GNRFA	<0.05	Davis et al. [23], Hong et al. [26]
Analgesic Use	Non-opioid Use	3 - 6 Months	Greater Reduction	Moderate Reduction	GNRFA	0.02 - 0.03	Davis et al. [23]
Safety Outcomes	Adverse Event Monitoring	Overall	Low	Low	Similar	-	Davis et al. [23], Sari et al. [25], Hong et al. [26], Badawy et al. [24]

Risk of Bias Assessment

**Figure 2:**
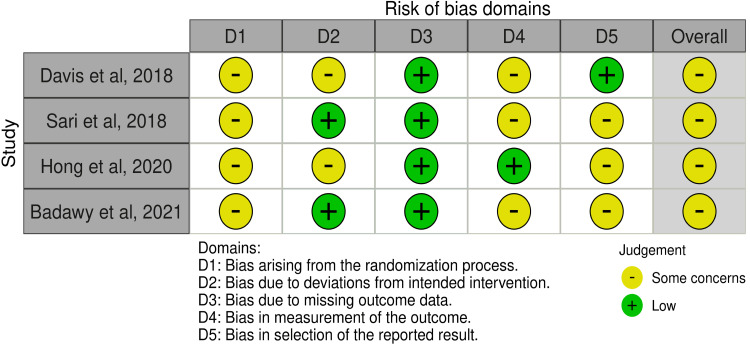
Visual Representation of the Risk of Bias in Selected Studies Studies included: Davis et al. (2018) [23], Sari et al. (2018) [25], Hong et al. (2020) [26], Badawy et al. (2021) [24].

The risk of bias in all four selected studies was assessed using the Cochrane RoB-2 tool. The overall assessment indicated some concerns. A summary of the findings is shown in Figure 2.

Discussion

This systematic review provides a more expansive analysis than previous reviews, which compared the effectiveness of GNRFA and IASI in the management of knee osteoarthritis [18]. This study conducted a broader database search, screened more studies, and extracted more clinical outcomes data than prior comparative reviews. Uniquely, this review also performed a formal assessment of statistical heterogeneity and calculated effect sizes using standardized mean differences, providing a more rigorous quantitative evaluation of the available data and a more comprehensive assessment of these two widely used interventions for patients with knee osteoarthritis.

Pain Outcomes

Across all four included studies, both GNRFA and IASI significantly reduced pain and improved functional outcomes when compared with baseline [23-26]. These findings reaffirm that both interventions are effective in managing knee osteoarthritis. However, distinct differences emerged in the timing and duration of therapeutic effect, favoring GNRFA for sustained long-term benefit. IASI provided a more rapid onset of improvement, with significantly superior pain reduction scores at one week [26]. In contrast, GNRFA demonstrated superior durability. At one month, three studies [23-25] showed a greater reduction in pain in the GNRFA group compared with IASI. This superiority of GNRFA in pain reduction continued to strengthen at three and six months, during which the IASI group consistently demonstrated waning therapeutic effects, approaching baseline symptom levels [23,24]. The effect size data support this pattern, as the SMD favoring GNRFA grew from -0.398 to -1.504 for Davis et al. [23], and from -0.844 to -0.437 for Badawy et al. [24] between one and six months, reflecting a large and increasing treatment effect for GNRFA over time [23,24]. Also, at six months, 22% of participants in the GNRFA group reported a complete absence of pain, while only 4% of participants in the IASI group reported a complete absence of pain [23]. Notably, compared with baseline, no worsening of pain was observed in the GNRFA group at six months, whereas 15% of patients in the IASI group reported worsening symptoms.

Functional Outcomes

Functional outcomes closely mirrored pain patterns. Although IASI demonstrated superior function at one week [26], GNRFA showed progressively better functional outcomes at one, three, and six months based on OKS and WOMAC function scores [23,24]. The effect size data support this pattern, as the SMD favoring GNRFA grew from 0.438 to 1.530 for Davis et al. [23], and from -0.122 to -0.620 for Badawy et al. [24] between one and six months, reflecting a large and increasing treatment effect for GNRFA over time. Similarly, the effect size data from Badawy et al. [24] demonstrated a growing effect favoring GNRFA, with the SMD increasing from a negligible and non-significant effect of -0.122 (p = 0.642) at one month to a large and statistically significant effect of -1.217 (p < 0.0001) at three months, and remaining moderate and statistically significant at -0.620 (p = 0.021) at six months, further supporting the sustained functional superiority of GNRFA over IASI. Also, a single exception was noted in the WOMAC stiffness subscale, where IASI demonstrated a statistically significant improvement at three months, while GNRFA showed no significant reduction in stiffness at this time point [23].

Patient Satisfaction

Patient satisfaction strongly favored GNRFA [23,26]. At three and six months, 80% and 91% of participants treated with GNRFA reported improvement in their knee osteoarthritis, compared with only 31% and 24% of those treated with IASI, respectively [23].

Analgesic Use

Analgesic usage patterns provided additional real-world insight. Opioid use remained unchanged in either group [23], suggesting that neither intervention substantially impacts patients requiring opioid use for their knee osteoarthritis. Non-opioid medication use significantly decreased in the GNRFA group, while the IASI group increased their use of non-opioid analgesics at three and six months [23]. This highlights GNRFA’s potential to reduce dependence on adjunctive pain medication over time.

Safety Profile

Both interventions demonstrated favorable safety profiles in all four studies [23-26]. No SAEs related to either treatment were reported; only minor, self-limiting, periprocedural discomfort was reported in one study [26]. These findings show that both GNRFA and IASI are safe and well-tolerated. The comparable safety profile suggests that treatment decisions can be based primarily on efficacy considerations rather than differential risk concerns.

Clinical Implications

Understanding the expected duration of benefit from each intervention provides important context for evaluating the findings of this review. Prior studies suggest GNRFA typically provides consistent pain control for three to six months [14,27], followed by a gradual decline in efficacy over the subsequent 6 to 12 months [27]. Pain recurrence is attributed to the regeneration of the ablated genicular nerves [14]. Comparatively, IASI typically provides pain relief lasting for about two to six weeks [28], and symptoms usually return to baseline levels within three to six months [29].

The findings of this review offer some clinical implications. For patients seeking rapid symptomatic relief, IASI may be preferred, given its superior early effects. However, for sustained pain control and functional improvement, GNRFA appears to be the superior option, maintaining therapeutic benefits through at least six months.

Interestingly, IASI may be a better choice for patients with knee stiffness, as it showed statistically significant improvement in WOMAC stiffness scores at three months, compared to GNRFA [25].

Several corticosteroids are utilized for knee IASI. Each corticosteroid differs in its pharmacology and chondrotoxic potential. In the included studies, betamethasone, methylprednisolone, and triamcinolone were used. In terms of clinical outcomes, these three corticosteroids have been reported to provide similar pain relief [30] and functional improvement [31]. Of these three corticosteroids, triamcinolone is associated with the greatest potential for chondrotoxicity [32]. Given that IASI effects typically diminish within weeks to a few months, to maintain the therapeutic effect, patients often require repeated injections. This raises concerns about the long-term safety of cumulative steroid exposure to the articular cartilage. Frequent steroid exposure has been associated with cartilage loss, further joint space narrowing, subchondral fractures, and osteoarthritis progression [33]. In contrast, GNRFA durability may reduce the need for repeated interventions.

Future Research

Future research should focus on larger, multicenter, randomized controlled trials directly comparing GNRFA and IASI. Additionally, longer-term follow-up studies are needed to establish the durability of GNRFA beyond six months and to determine the optimal timing for repeat interventions. Also, studies examining the patient subgroup most likely to benefit from each intervention could enable clinicians to select treatments that are more personalized for each patient. Additionally, the role of combination or sequential GNRFA and IASI therapy also merits investigation. Given that IASI has a rapid onset and GNRFA has a sustained effect, sequential therapy might optimize both immediate symptom control and long-term management.

Limitations

While this systematic review provides valuable insights, it has some limitations, including only four randomized controlled trials meeting the inclusion criteria, variation in GNRFA techniques (cooled and conventional), variation in IASI composition, substantial heterogeneity across studies, and some studies having some concern in the risk-of-bias assessment. These factors may influence cross-study comparisons but do not diminish the consistency of overall findings.

## Conclusions

This systematic review of four randomized controlled trials involving 379 patients demonstrates that both GNRFA and IASI are effective interventions for knee osteoarthritis, but they differ in their effectiveness. IASI provided superior pain relief and functional improvement within the first week after treatment, but its effects steadily declined over the next three to six months. In contrast, GNRFA demonstrated superior and sustained therapeutic benefits from one through six months across multiple domains, including sustained pain reduction, higher patient satisfaction, and decreased reliance on non-opioid analgesics. Both interventions demonstrated favorable safety profiles, with minimal AEs.

Based on these findings, GNRFA appears to be the preferred intervention for patients seeking durable pain control and functional improvement in knee osteoarthritis, while IASI remains a valuable option for patients requiring rapid symptomatic relief or those with prominent joint stiffness. Given the small number of identified studies, the findings of this review should be generalized with caution. Treatment selection should be individualized through shared decision-making that considers patient preferences, symptom characteristics, and the anticipated timeline for therapeutic benefit. Future research should focus on longer-term comparative effectiveness studies, the identification of patient subgroups most likely to benefit from each intervention, and the evaluation of combination or sequential treatment strategies to further optimize the management of knee osteoarthritis.

## References

[1] Steinmetz JD, Culbreth GT, Haile LM (2023). Global, regional, and national burden of osteoarthritis, 1990-2020 and projections to 2050: a systematic analysis for the Global Burden of Disease Study 2021. Lancet Rheumatol.

[2] Zhang Y, Jordan JM (2010). Epidemiology of osteoarthritis. Clin Geriatr Med.

[3] Coaccioli S, Sarzi-Puttini P, Zis P, Rinonapoli G, Varrassi G (2022). Osteoarthritis: new insight on its pathophysiology. J Clin Med.

[4] Leifer VP, Katz JN, Losina E (2022). The burden of OA-health services and economics. Osteoarthritis Cartilage.

[5] Yusuf E (2016). Pharmacologic and non-pharmacologic treatment of osteoarthritis. Curr Treatm Opt Rheumatol.

[6] Osuala U, Goh MH, Mansur A (2024). Minimally invasive therapies for knee osteoarthritis. J Pers Med.

[7] Warren BJ, Fleeks JL, Bhardwaj N (2023). Current trends in intra-articular knee injections among family physicians. PRiMER.

[8] Pirri C, Sorbino A, Manocchio N, Pirri N, Devito A, Foti C, Migliore A (2024). Chondrotoxicity of intra-articular injection treatment: a scoping review. Int J Mol Sci.

[9] Saha P, Smith M, Hasan K (2023). Accuracy of intraarticular injections: blind vs. image guided techniques - a review of literature. J Funct Morphol Kinesiol.

[10] Choi WJ, Hwang SJ, Song JG, Leem JG, Kang YU, Park PH, Shin JW (2011). Radiofrequency treatment relieves chronic knee osteoarthritis pain: a double-blind randomized controlled trial. Pain.

[11] Jamison DE, Cohen SP (2018). Radiofrequency techniques to treat chronic knee pain: a comprehensive review of anatomy, effectiveness, treatment parameters, and patient selection. J Pain Res.

[12] Koshi E, Meiling JB, Conger AM, McCormick ZL, Burnham TR (2022). Long-term clinical outcomes of genicular nerve radiofrequency ablation for chronic knee pain using a three-tined electrode for expanded nerve capture. Interv Pain Med.

[13] Desai M, Bentley A, Keck WA, Haag T, Taylor RS, Dakin H (2019). Cooled radiofrequency ablation of the genicular nerves for chronic pain due to osteoarthritis of the knee: a cost-effectiveness analysis based on trial data. BMC Musculoskelet Disord.

[14] Kidd VD, Strum SR, Strum DS, Shah J (2019). Genicular nerve radiofrequency ablation for painful knee arthritis: the why and the how. JBJS Essent Surg Tech.

[15] Kapural L, Minerali A, Sanders M, Matea M, Dua S (2022). Cooled radiofrequency ablation provides prolonged pain relief compared to traditional radiofrequency ablation: a real-world, large retrospective clinical comparison from a single practice. J Pain Res.

[16] Karaman H, Tüfek A, Kavak GÖ, Yildirim ZB, Uysal E, Celik F, Kaya S (2011). Intra-articularly applied pulsed radiofrequency can reduce chronic knee pain in patients with osteoarthritis. J Chin Med Assoc.

[17] Chou SH, Shen PC, Lu CC (2021). Comparison of efficacy among three radiofrequency ablation techniques for treating knee osteoarthritis: a systematic review and meta-analysis. Int J Environ Res Public Health.

[18] Chalidis B, Papadopoulos P, Givissis P, Pitsilos C (2023). Is radiofrequency ablation superior to intra-articular injections for the treatment of symptomatic knee osteoarthritis? A systematic review. J Pers Med.

[19] Moher D, Liberati A, Tetzlaff J, Altman DG (2009). Preferred reporting items for systematic reviews and meta-analyses: the PRISMA statement. PLoS Med.

[20] Babineau J (2014). Product review: Covidence (systematic review software). J Can Health Libr Assoc.

[21] Sterne JA, Savović J, Page MJ (2019). RoB 2: a revised tool for assessing risk of bias in randomised trials. BMJ.

[22] McGuinness LA, Higgins JP (2021). Risk-of-bias VISualization (ROBVIS): an R package and Shiny web app for visualizing risk-of-bias assessments. Res Synth Methods.

[23] Davis T, Loudermilk E, DePalma M (2018). Prospective, multicenter, randomized, crossover clinical trial comparing the safety and effectiveness of cooled radiofrequency ablation with corticosteroid injection in the management of knee pain from osteoarthritis. Reg Anesth Pain Med.

[24] Badawy FA, Mohamed AT, Abdelrahman AE, Mahmoud WA (2021). Comparison of intraarticular injection versus radiofrequency neurotomy in knee osteoarthritis. Sohag Med J.

[25] Sarı S, Aydın ON, Turan Y, Özlülerden P, Efe U, Kurt Ömürlü İ (2018). Which one is more effective for the clinical treatment of chronic pain in knee osteoarthritis: radiofrequency neurotomy of the genicular nerves or intra-articular injection?. Int J Rheum Dis.

[26] Hong T, Li G, Han Z, Wang S, Ding Y, Yao P (2020). Comparing the safety and effectiveness of radiofrequency thermocoagulation on genicular nerve, intraarticular pulsed radiofrequency with steroid injection in the pain management of knee osteoarthritis. Pain Physician.

[27] Lyman J, Khalouf F, Zora K (2022). Cooled radiofrequency ablation of genicular nerves provides 24-month durability in the management of osteoarthritic knee pain: outcomes from a prospective, multicenter, randomized trial. Pain Pract.

[28] Bensa A, Albanese J, Boffa A, Previtali D, Filardo G (2024). Intra-articular corticosteroid injections provide a clinically relevant benefit compared to placebo only at short-term follow-up in patients with knee osteoarthritis: a systematic review and meta-analysis. Knee Surg Sports Traumatol Arthrosc.

[29] Jüni P, Hari R, Rutjes AW, Fischer R, Silletta MG, Reichenbach S, da Costa BR (2015). Intra-articular corticosteroid for knee osteoarthritis. Cochrane Database Syst Rev.

[30] Wattanasirisombat K, Boontanapibul K, Pinitchanon P, Pinsornsak P (2026). Betamethasone and triamcinolone acetonide have comparable efficacy as single intra-articular injections in knee osteoarthritis: a double-blinded, randomized controlled trial. J Bone Joint Surg Am.

[31] Lomonte AB, de Morais MG, de Carvalho LO, Zerbini CA (2015). Efficacy of triamcinolone hexacetonide versus methylprednisolone acetate intraarticular injections in knee osteoarthritis: a randomized, double-blinded, 24-week study. J Rheumatol.

[32] Fackler NP, Yareli-Salinas E, Callan KT, Athanasiou KA, Wang D (2023). In vitro effects of triamcinolone and methylprednisolone on the viability and mechanics of native articular cartilage. Am J Sports Med.

[33] Ibad HA, Kasaeian A, Ghotbi E (2023). Longitudinal MRI-defined cartilage loss and radiographic joint space narrowing following intra-articular corticosteroid injection for knee osteoarthritis: a systematic review and meta-analysis. Osteoarthr Imaging.

